# The Afghan migrant crisis: An impending threat to Pakistan's health system

**DOI:** 10.1016/j.amsu.2022.104180

**Published:** 2022-07-14

**Authors:** Abdullah Nadeem

**Affiliations:** Department of Medicine, Dow University of Health Sciences, Karachi, Pakistan

Pakistan is facing an impending crisis due to mass Afghan migrations after almost four decades as the political and social framework in Afghanistan falls apart. There has been a persistent influx of Afghan people seeking safety, sanctuary, and occasionally business opportunities in Pakistan due to the ongoing war in their country. With most prevailing diseases of Pakistan on rampant and COVID-19 pandemic engulfing the globe, is Pakistan ready to deal with diseases associated with mass migrations?

Even in ideal circumstances, migration includes a sequence of incidents that can be extremely traumatic and put migrants at risk [1]. Disease burden associated with international movements include transmissible infections. They result in introduction of non-endemic infectious strains and increase the prevalence of certain diseases in the host population [2]. Pakistan's endemic infectious diseases are an important cause of morbidity and mortality, and we believe it cannot afford any increase in the same [3].

Mass migration leads to transmission of various infectious diseases including malaria, typhoid, TB, hepatitis B and dysentery. Pakistan and Afghanistan remain the only two countries in the world which have failed to eradicate the polio virus completely. An influx of Afghan refugees would not only cause increased transmission, but disease spread from unregistered migrants would also create discrepancies in statistics, cause underestimation of infected cases and improper tracing to index cases.

Migrants often live with minimum utilities, and face social discrimination, as a result they often find places in lower strata of society and face unemployment resulting in malnutrition and starvation [4]. A 2004 study found out the long-term complications of starvation, which included cardiovascular diseases and diabetes mellitus [5]. Pakistan already experiences its fair share of cardiovascular diseases whereby a study estimated that 1 in 4 middle aged Pakistani adults suffer from coronary artery disease [6]. Also, according to the CDC ischemic heart disease is the number one cause of mortality in Pakistan [7].

Refugees from war-stricken countries inevitably come with their past traumas, most commonly associated with psychological health problems especially amongst children and mothers. The most common disorders diagnosed include post-traumatic stress disorders, major depression and generalized anxiety [8]. In Pakistan mental disorders already make up 4% of the disease burden with 24 million people believed to be in need of psychiatric consultation [9].

We think with a mass migration Pakistan's healthcare system would be tested in its most weak areas, especially amidst the pandemic. This then might result in economic strains and compromises on the health and well-being of native Pakistanis as well as the migrant refugees.

Hence, it would be essential for Pakistan to work collaboratively with resource rich countries and different local and international humanitarian relief organizations to curb the Afghan migrations in order to prevent any economic, health and humanitarian crisis.

## Ethical approval

This paper did not involve patients; therefore, no ethical approval was required.

## Funding

No funding was acquired for this paper.

## Author contribution

Abdullah Nadeem: conception of the study, drafting of the work, final approval and agreeing to the accuracy of the work.

## Guarantor

Abdullah Nadeem.

## Consent

This study was not done on patients or volunteers, therefore no written consent was required.

## Declaration of competing interest

The author declares that there is no conflict of interest.
